# Intronic Variants of the Angiotensin-Converting Enzyme 2 Gene Modulate Plasma ACE2 Levels and Possibly Confer Protection against Severe COVID-19

**DOI:** 10.1155/2023/5705076

**Published:** 2023-10-26

**Authors:** Rubaiat Ahmed, Abdullah Al Saba, Anik Paul, Jasmin Nur, Md Sohrab Alam, Sajib Chakraborty, Md. Zakir Hossain Howlader, Laila N. Islam, A. H. M. Nurun Nabi

**Affiliations:** ^1^Laboratory of Population Genetics, Department of Biochemistry and Molecular Biology, University of Dhaka, Dhaka 1000, Bangladesh; ^2^Department of Immunology, Bangladesh Institute of Research and Rehabilitation in Diabetes, Endocrine and Metabolic Disorders, Shahbag, Dhaka, Bangladesh; ^3^Laboratory of Nutrition and Health Research, Department of Biochemistry and Molecular Biology, University of Dhaka, Dhaka 1000, Bangladesh

## Abstract

Membrane-bound angiotensin-converting enzyme 2 (*ACE2*) receptor acts as the entry point for the novel coronavirus, SARS-CoV-2. Polymorphisms in the *ACE2* gene may alter viral binding, regulate the expression of ACE2, and thus, affect disease severity. In this study, 68 COVID-19 patients with varying degrees of severity and 40 healthy controls were enrolled. The genetic landscape of the *ACE2* gene was explored by whole exome sequencing of 29 individuals, and specific regions of *ACE2* were analyzed for the rest of the participants via PCR, followed by barcode-tagged sequencing. The mean soluble ACE2 level in the plasma of healthy controls and patients did not vary significantly but was higher in the patient group (3.77 ± 1.55 ng/mL vs. 3.94 ± 1.42 ng/mL). Analysis of exon 1, exon 2, and exon 8 of the *ACE2* gene revealed that these regions are highly conserved in our population. Investigation of exon 11 and its flanking intronic region revealed that deletions in a stretch of 18T nucleotides in the noncoding region significantly decrease ACE2 levels in plasma, as individuals harboring wild-type variants had higher plasma ACE2 levels compared to those harboring T1del, T2del, and T3del variants. However, the intronic variants were not found to be significantly associated with disease severity.

## 1. Introduction

Since its emergence in December 2019, the severe acute respiratory syndrome coronavirus 2 (SARS-CoV-2), known as the novel coronavirus, has caused a global pandemic named coronavirus disease 2019 (COVID-19). To date, the virus has caused over 696 million infections worldwide, killing more than 6.9 million people (https://www.worldometers.info/coronavirus/). The virus's ability to cause asymptomatic infections and viral shedding from asymptomatic individuals has enabled the virus to spread at a greater speed throughout the world [1].

The virus consists of a 29.9 kb positive-sense single-strand RNA (+ssRNA), packed by one of the 4 structural proteins, the nucleocapsid (N) protein, inside an envelope. The envelope contains the remaining three structural proteins: membrane protein (M), spike protein (S), and envelope protein (E). The virus also produces sixteen nonstructural proteins (nsp1−16) which perform the necessary actions for the virus to take over the host cells and replicate successfully [2]. SARS-CoV-2 gains entry into the host cells by interaction of the S protein and the angiotensin-converting enzyme 2 (ACE2) receptor of the lung epithelium and alveolar type II pneumocytes [3, 4]. The proper interaction between these two proteins is of paramount importance. As the virus replicates through the action of an RNA-dependent RNA polymerase (RdRP) enzyme, it harbors many mutations in the genome [5]. Retaining these mutations gives rise to variants of the virus, which brings changes to its characteristics, such as infectivity, transmissibility, replication capability, and adverse immune responses [6].

*ACE2* gene is located on the X chromosome that spans approximately 96 kb of genomic DNA and contains 18 exons [7]. ACE2 is ubiquitously expressed in different parts of the human body. Abundant localization of ACE2 has been found in the epithelia of the lungs and intestine [8]. ACE2-dependent entry of SARS-CoV-2 through its spike protein is a critical step of the infection [9]. ACE2 is a member of the renin-angiotensin system (RAS), and it plays a major role in the regulation of blood pressure [10]. Thus, the dual functionality of ACE2, as a blood pressure regulator and as the receptor for SARS-CoV-2, gives strong motivation to the researchers behind the cause of disease severity in individuals with comorbidity especially hypertension and diabetes [11]. Single nucleotide polymorphisms (SNPs) of the *ACE2* gene have been studied in different populations to find out the relationship with diseases with incompatible results. SNPs of *ACE2* have also been studied to find the association with essential hypertension, dyslipidemia, hypertrophic cardiomyopathy, ventricular hypertrophy, and cerebral malaria [12–14]. Polymorphisms within the *ACE2* gene have been identified to be associated with impaired activity of ACE2, thus resulting in an abnormal level of its product, angiotensin (1-7) [9].

SARS-CoV-2 can cause asymptomatic, mild, moderate, and severe forms of infection [15]. During the pandemic, even the same variant of the virus caused different degrees of disease severity in different populations all over the world [16]. Many reasons can be attributed to this spectrum of disease severity, such as environmental factors [17], immunogenetics [18], and also economic development level [19]. However, even in the most densely populated countries like Bangladesh, the devastating impacts of this virus were not seen as prominent as it was observed in the most developed regions of the world, particularly in the European countries, as demonstrated by total cases and deaths per 1 million population (https://www.worldometers.info/coronavirus/). Thus, other than the causes mentioned above, host genetic factors must play an important role.

Incompatible results have been reported regarding the association of *ACE2* gene variants with the risk as well as the severity of COVID-19 disease. Studies conducted on Turkish, Italian, and Spanish populations reported that *ACE2* gene rs2106809 and rs2285666 polymorphisms were not associated with the severity of COVID-19 infection [20–22], while Sienko et al. demonstrated that genotypes AA, TT, GG, TT, and TT, respectively, of rs2285666, rs2074192, rs4646174, rs4646156, and rs2158083 of the *ACE2* gene have the most significant correlation with COVID-19 in Polish population [23]. On the other hand, in another study, Cafiero et al. reported that SNPs within the members of the renin-angiotensin system such as rs2074192 within *ACE2*, rs1799752 within *ACE*, and rs699 within *angiotensinogen* (*AGT*) could potentially be a valuable tool for predicting the clinical outcome of SARS-CoV-2-infected patients [24].

Thus, it is indeed very important to understand the disease severity from the point of view of host genetics, i.e., the impact of variants within the *ACE2* gene on the COVID-19 disease severity. Suryamohan et al. demonstrated that the missense variants of ACE2 protein S19P, I21V, E23K, K26R, T27A, N64K, T92I, Q102P, and H378R were predicted to increase disease susceptibility while K31R, N33I, H34R, E35K, E37K, D38V, Y50F, N51S, M62V, K68E, F72V, Y83H, G326E, G352V, D355N, Q388L, and D509Y were predicted to be protective that show decreased binding to S protein [25]. Recently, we demonstrated that the most frequently harbored missense variants of *ACE2* in different populations show different patterns of binding with the S protein of different SARS-CoV-2 variants [26]. Thus, in the present study, we aimed to (i) reveal the landscape of the polymorphic patterns within the exons of the *ACE2* gene, (ii) evaluate the association of flanking intronic variants with COVID-19 disease severity, (iii) study the relationship between plasma ACE2 levels and COVID-19 severity, and (iv) explore the roles of the *ACE2* variants on plasma ACE2 levels.

## 2. Materials and Methods

### 2.1. Study Design and Sample Collection

The study was approved by the Ethical Review Committee of the Faculty of Biological Sciences, University of Dhaka. A total of 68 SARS-CoV-2 infected patients and 40 healthy individuals were enrolled. Individuals who tested positive for SARS-CoV-2 by RT-PCR were considered COVID-19 patients.

Samples were collected from the COVID-19 unit of BIRDEM General Hospital, Dhaka, Bangladesh, and, locally, after getting full consent from the patients and/or their closely related attendants during August and October 2021. Depending on the nature of their symptoms, biochemical parameters, oxygen saturation, and intensive care unit (ICU) requirements, the patients were classified according to disease severity, as suggested by Yuki et al. [27]. Out of the total infected patients, 24 had mild, 20 had moderate, and 24 had severe symptoms due to COVID-19 disease. The average oxygen saturation of mild patients was 97.4% during the day of sampling for the RT-PCR test, and they were not admitted to the hospital. Patients with moderate symptoms were hospitalized with an average oxygen saturation of 94.71% at the time of admission but did not require oxygen. The average oxygen saturation in severe patients was below 88.4%, and they were admitted to the ICU. Mild patients had complaints of abdominal pain, acute gastroenteritis, cough, mild fever, body pain, and headache. Moderate patients also had body pain, cough, mild fever, headache, muscle aches, sore throat, runny nose, history of hypertension, diabetes, osteoarthritis, chronic kidney disease, and chronic obstructive pulmonary disease. Along with these symptoms, severe patients had a history of ischemic heart disease, stroke, long-term diabetes and hypertension, myocardial infarction, chronic renal failure, chronic liver disease, and more than 15% lung involvement (confirmed by CT scan report).

Control samples were collected from healthy individuals who had no record of SARS-CoV-2 infection. They had no record of fever for at least 30 days before sampling, and no records of cough and asthma were reported.

Three (3.0) milliliters of blood samples were collected in the EDTA-containing vacutainer tube from the study participants. The collected samples were transported to the Laboratory of Population Genetics, Department of Biochemistry and Molecular Biology, University of Dhaka, using an ice box with appropriate precautionary measures. The subsequent experiments and procedures were performed in a negative air pressure laboratory environment, using protective equipment to prevent contamination by infectious samples. While performing the experimental procedures, the sample categories (both the healthy control and patients with different severity classes) were not identifiable. After the experiments were done, during the analysis of the data, the identity of all groups was revealed. Plasma samples were separated through centrifugation at 6000 rpm for 5 minutes, and along with the cellular part of the samples, plasma-containing tubes were stored at -80°C till further analysis.

A structured questionnaire was prepared to record the demographic information and the biochemical parameters of each study participant. Information about the age, gender, oxygen saturation, degree of severity of the disease, CT scan report on lung involvement due to SARS-CoV-2, and comorbidities like diabetes, hypertension, cardiovascular diseases, ischemic heart disease, myocardial infarction, chronic obstructive pulmonary disease, and chronic kidney disease was recorded, and data regarding biochemical markers like levels of serum ferritin, D-dimer, and C-reactive protein (CRP) were measured.

### 2.2. Determination of Soluble ACE2 in the Plasma of the Study Participants

The enzyme-linked immunosorbent assay (ELISA) against the human ACE2 was performed using the ab235649 Human ACE2 SimpleStep ELISA® Kit (Abcam, United Kingdom). The kit uses a combination of three antibodies (an anti-tag antibody coating the well, an affinity tag labeled capture antibody, and a reporter-conjugated detector antibody) to immobilize the ACE2 via immunoaffinity on the well. The detector antibody uses horse radish peroxidase (HRP) as the reporter enzyme. The kit has an intra-assay coefficient of variation (CV%) of 2.3%, and in the case of interassay, it is 3.2%. The sensitivity of this kit is 1052 pg/mL.

The protocol provided by the manufacturer was followed in all steps of the assay. At first, an antibody cocktail was prepared, combining the capture and detector antibodies in the supplied antibody diluent. To prepare the standard curve, the lyophilized ACE2 provided by the supplier was reconstituted using the sample diluent normal saline to a concentration of 4080 ng/mL. Then, a serial dilution was prepared from the stock solution which ranged from 255 ng/mL to 0 ng/mL (blank). The samples were diluted by adding an equal amount of plasma and sample diluent normal saline to obtain a 1 : 2 ratio.

The wells were coated with the anti-tag antibody. The samples were added to the wells, as well as the standards. Then, the antibody cocktail was added to each well. The plates were sealed and incubated for 1 hour at room temperature on a plate shaker set at 400 rpm. After incubation, the plates were washed with the provided wash buffer 3 times. It was made sure that any excess liquid was removed at each step. As coloring reagent, 3,3′,5,5′-tetramethylbenzidine (TMB) was added to the wells and incubated under the same condition. After 10 minutes, the stop solution was added. The optical density (OD) was measured at 450 nm. The calculations were done by constructing a standard curve, as per the manufacturer's instructions.

### 2.3. Whole Exome Sequence Analyses

DNA was extracted from the cellular fraction of the blood samples in an organic method employing EDTA (0.5 M, pH 8.0), Tris-HCl (1 M, pH 7.6), red blood cell lysis buffer (1 M Tris, sucrose, and MgC1_2_, pH 8.0), Triton X-100, and SDS, as reported in our previous studies [28–30], and the quantity and quality of the extracted DNA were measured using NanoDrop One^C^ Microvolume UV-Vis Spectrophotometer (Thermo Fisher Scientific, US). The ratio of the absorbances observed at 260 nm and 280 nm (A260/280) and also at 260 nm and 230 nm (A260/230) was used as the measurement of quality, and DNA samples having values ~1.8 for A260/280 and 2.0-2.2 for A260/230 were considered pure.

Extracted DNA samples from 22 blood samples with COVID-19 disease (severe = 8, moderate = 7, and mild = 7) and 7 blood samples of healthy individuals with no record of SARS-CoV-2 infection were used for the whole exome analysis. Whole exome sequencing was conducted from the DNA samples using the NovaSeq 6000 sequencing platform that uses Illumina SBS technology. The sequencing library was prepared using the Twist Human Core Exome library preparation kit. The protocol that was followed is the Twist Human Core Exome Sequencing preparation guide. The coverage of Twist Human Core Exome is greater than 99% (33.05 Mb human coding regions) of protein-coding genes. The DNA sequences were assembled and aligned to reference gene sequences based on the human genome build GRCh38/UCSC hg38 and analyzed.

### 2.4. Primer Design, Polymerase Chain Reaction, and Sequencing of the Exons

Individual regions of the exon sequences were retrieved from NCBI (reference sequence: NG_012575.3). Primer3 web-based tool was used to design primers to amplify each exonic region. Three pairs of primers harboring exonic regions of exon 1, exon 2, and exon 8 were used to amplify regions of interest (as shown in Supplementary Figure 1). The primer sequences are provided in Supplementary Table 1. Polymerase chain reaction (PCR) for each primer set to amplify respective exons was performed, followed by purification of each product. The PCR conditions are provided in Supplementary Table 2. Barcode-tagged sequencing (BTSeq) was done to find out the landscape of each exon of the *ACE2* gene and find mutational hotspots. Chromatograms were analyzed using Geneious 11.1.5 (https://www.geneious.com). The sequences of each individual were aligned with the reference nucleotide sequence of the *ACE2* gene of Homo sapiens (NCBI accession: NG_012575.3).

### 2.5. Amplification and Sequencing of the Noncoding Region including the 18T Stretch

Flanking sequences of the exonic regions analyzed from the whole exome data revealed that frequencies of deletion of “T” nucleotide(s) within a stretch of 18Ts residing upstream of exon 11 located from 15573562 to 15573579 of the reference sequence of the X chromosome (GRCh38.p13 Chr X) varied in patients with different degrees of disease severity. Thus, this region was amplified by PCR using a distinct primer pair to have 900 bp amplicons (as shown in Supplementary Figure 2) which were purified, and then, sequencing was performed using the BTSeq method. The primer sequences are provided in Supplementary Table 1, and the PCR condition is shown in Supplementary Table 2.

### 2.6. Statistical Analyses

Statistical analyses were conducted using R programming language (version 4.1.2). Categorical variables (genetic variants and disease severity classes) were summarized as percentage, and the continuous variables (age, biochemical parameters and plasma ACE2 level) were expressed as mean ± SD. The significance of the difference in mean plasma ACE2 levels between different groups was measured using the Welch two-sample *t*-test and one-way analysis of variance (ANOVA) (for comparison between more than 2 groups). As a post hoc analysis, Tukey's honest significance test was done. The association between different groups and disease severity was measured using Fisher's exact test. The odds ratio (OR) of risk was calculated at a 95% confidence interval (CI) for each variant (T1del, T2del, and T3del) and all variants combined, against the wild type (18T) to measure the odds of different degrees of disease severity occurring due to different variants on the 18T stretch. Pearson's correlation coefficient was used to assess the relationship between age and plasma ACE2 level, and Spearman's rank correlation coefficient was used to evaluate the correlation of plasma ACE2 level with disease severity. The plots were generated with the ggplot2 package, and the remaining calculations were done using the dplyr and epitools packages in R [31] [32]. A *p* value of less than 0.05 was considered statistically significant.

## 3. Results

### 3.1. Demographic and Biochemical Information of the Study Participants

Out of the total study participants (*n* = 108), 49 were female, and 59 were male. And in the patient group (*n* = 68), there were 31 females and 37 males. The demographic and biochemical data of enrolled patients are summarized in Table 1.

It is observed from Table 1 that the mean CRP level is significantly different between patients with different degrees of disease severity, with the moderate group having the highest level (92.07 ± 57.41 mg/L). Male patients had a significantly higher mean CRP level (72.29 ± 41.09 mg/L) than female patients. The oxygen saturation level was also significantly different between the severity classes, with the severe group having the lowest saturation level (88.4 ± 5.26%) and the mild group having the highest saturation (97.4 ± 1.52%).

### 3.2. Whole Exome Sequencing (WES) and Identification of Mutational Hotspot

**Figure 1:**
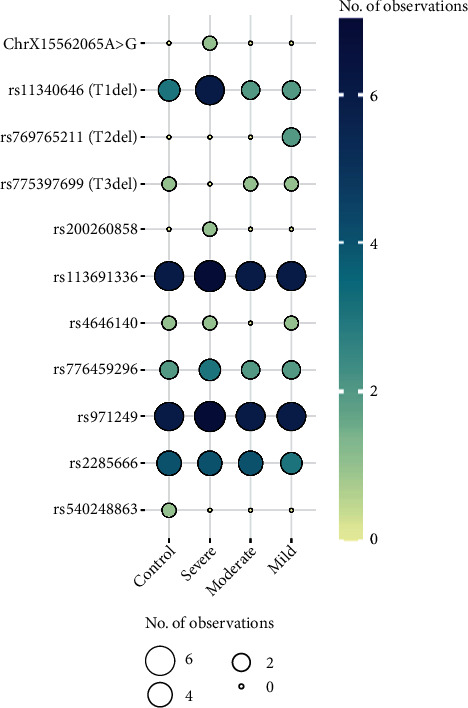
Variants of the *ACE2* gene identified by WES and their number of occurrences in samples of different disease severity classes. The size of the circle and shade of blue represent the number of occurrences. A larger circle and a darker shade of blue indicate a greater number of occurrences.

The whole exome sequencing revealed the genetic landscape of the *ACE2* gene in the selected samples. The exonic regions were fully conserved in all 29 individuals; that is, no synonymous or nonsynonymous variants were found within the three exons of interest (exon 1, exon 2, and exon 8) of ACE2 receptor that constitute the binding regions for the S protein of SARS-CoV-2. However, there were many variants observed in the flanking intronic regions. The results are summarized in Figure 1.

Three (3) individuals (one individual from the control, mild, and moderate groups each) harbored the reference ACE2 sequence; that is, they had no variants in the *ACE2* gene.

The observed allele frequency in our dataset and those reported in different databases are shown in Table 2.

The variants rs11340646, rs769765211, and rs775397699 all occur at an 18T stretch on chromosome X, which is just upstream of exon 11. rs113691336 and rs971249 are also highly prevalent in our study population, but their occurrence is fairly similar in all groups of participants (control, mild, moderate, and severe). That is why we decided to proceed with the 18T region of the *ACE2* gene including exon 11, along with exons 1, 2, and 8.

### 3.3. Polymerase Chain Reaction (PCR) and Amplicon Sequencing

Upon performing PCR reactions for the remaining 79 samples (33 controls and 46 samples), no variants were observed in exons 1, 2, and 8 either. Therefore, we can conclude that this particular region is highly conserved in our population, i.e., harboring neither synonymous nor nonsynonymous variants.

After analyzing exon 11 along with its upstream and downstream nucleotides (900 bp), it was observed that 4 (16.6%) severe patients, 4 (20.0%) moderate patients, 2 (8.3%) mild patients, and 5 (12.5%) healthy individuals harbored wild-type (WT) stretch of 18T nucleotides. Further, deletion of a single T (T1del) nucleotide from the stretch was found in 10 (41.7%) severely infected, 6 (30.0%) moderately infected, 3 (12.5%) mildly infected, and 15 (37.5%) healthy individuals; deletion of two T (T2del) nucleotides was found in 10 (41.7%) severely infected, 8 (40.0%) moderately infected, 17 (70.8%) mildly infected, and 19 (47.5%) of the healthy individuals; and deletion of three T (T3del) was found in 2 (10.0%) moderately infected, 2 (8.3%) mildly infected, and 1 (2.5%) of the healthy individuals while this particular type was not found in patients with severe symptoms.

### 3.4. Association of Genetic Variants with Disease Severity

No significant association was found between genetic variants occurring at the 18T stretch and disease severity (*p* = 0.186).

For odds ratio estimation, the control and mild phenotypes were considered protective, whereas moderate and severe phenotypes were taken as risk groups. The results are given in Table 3.

We also calculated the OR between WT and all variant groups, considering the previous stratification of protective and risk groups, and obtained similar results (OR = 0.56; 95% CI = 0.18‐1.71, *p* = 0.28).

Therefore, the deletions play a protective role against the development of moderate and severe COVID-19, as the OR is less than 1 in all cases. However, the results were not significant, as the *p* values are higher than 0.05.

### 3.5. Levels of ACE2 Measured in the Plasma of Study Participants

The ACE2 levels in the plasma of 72 samples (20 healthy controls, 22 mild patients, 11 moderate patients, and 19 severe patients) were measured. We calculated the mean plasma ACE2 levels and stratified them against different parameters.

**Figure 2:**
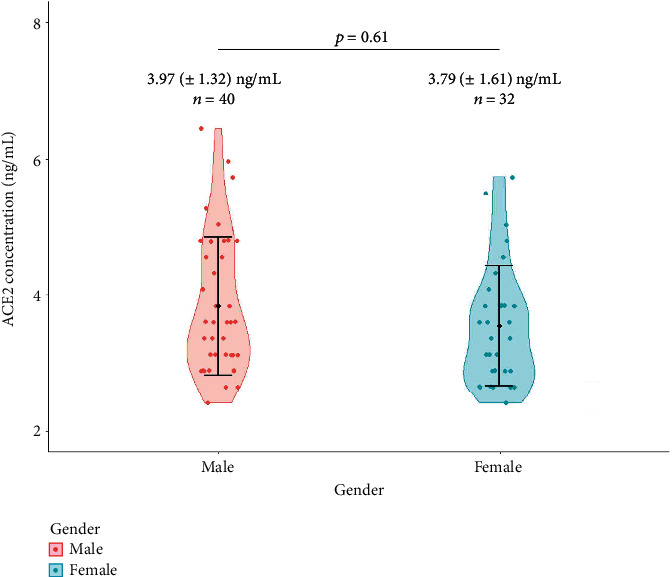
Violin plot showing the plasma ACE2 concentration in male and female participants. Male participants have higher plasma ACE2 level (indicated in red), compared to female participants (shown in blue). The black dots in the error bars correspond to the mean value in each group.

When stratified against gender, no significant difference was observed in the mean ACE2 level in male and female participants (*p* = 0.61). The results are shown in Figure 2.

**Figure 3:**
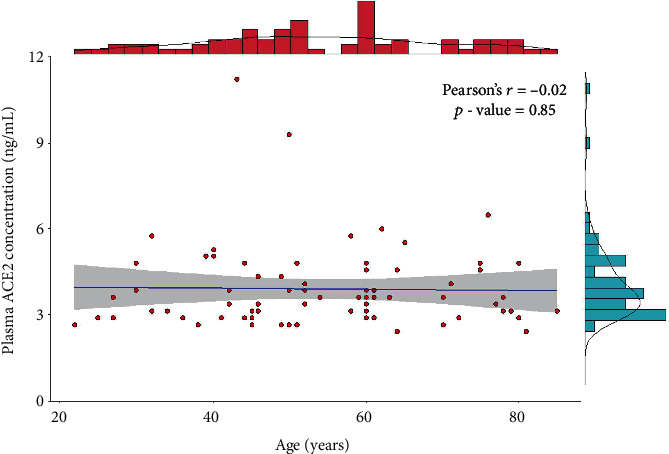
Scatterplot with marginal histogram demonstrating the distribution and correlation between plasma ACE2 levels and participant's age. Only a marginally negative correlation was observed (*r* = −0.02).

Male participants have higher plasma ACE2 level (indicated in red), compared to female participants (shown in blue), but the difference was not statistically significant. We also analyzed if age is correlated with ACE2 level. But no significant correlation was observed (*r* = −0.02, *p* = 0.86). Therefore, age and gender have not played any confounder role in our analyses. The correlation between age and plasma ACE2 level is shown in Figure 3.

**Figure 4:**
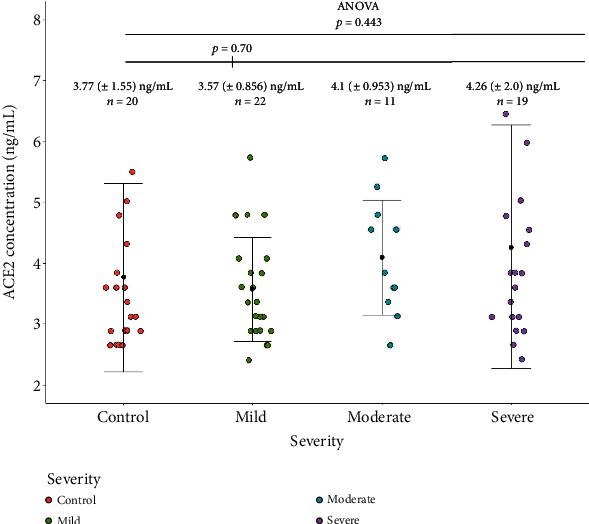
Jitter plot demonstrating the plasma ACE2 levels in healthy controls and patients with different degrees of COVID-19 disease severity. Severe patients have the highest plasma ACE2 level (shown in purple), and the mild group has the lowest (shown in green). The black dots within the error bars represent the mean ACE2 levels for each group.

We then proceeded to stratify plasma ACE2 levels against disease severity. The results are summarized in Figure 4.

The results indicate that the severe patients have the highest plasma ACE2 level (4.26 ± 2.0 ng/mL), and the mild group has the lowest (3.57 ± 0.856 ng/mL). But the mean ACE2 levels were not significantly different between the disease severity groups. When grouped together, it was observed that the plasma ACE2 level was higher in the patient group (3.94 ± 1.42 ng/mL), compared to the control group (3.77 ± 1.55 ng/mL), but the results were not significantly different either (*p* = 0.7). A weak positive correlation was found between the plasma ACE2 levels and disease severity (Rs = 0.18, *p* = 0.13).

**Figure 5:**
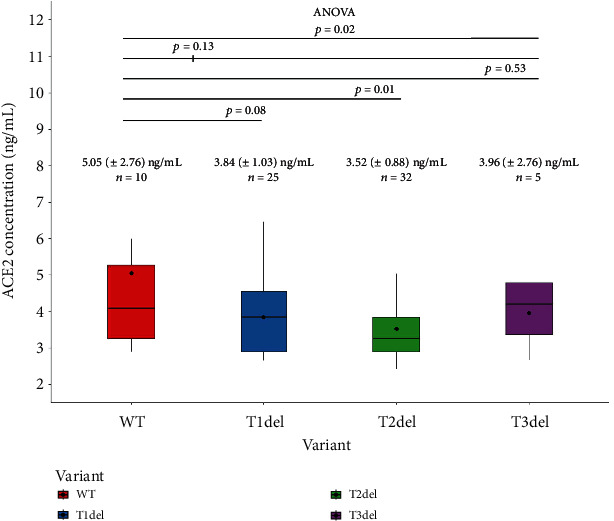
Boxplots showing the plasma ACE2 levels in individuals with WT and deletion variants of the 18T stretch. Individuals harboring deletion variants of the 18T stretch (T1del in blue, T2del in green, and T3del in purple) have lower plasma ACE2 levels, compared to the WT variant harboring individuals (shown in red). The black dots in each box represent the mean values. The black vertical lines indicate the range.

We observed significant difference (*p* = 0.02) in mean plasma ACE2 levels between individuals harboring different variants of the 18T stretch. The results are shown in Figure 5.

Individuals with the WT variant (18T) contain the highest plasma ACE2 level (5.05 ± 2.76 ng/mL), and the individuals harboring the T2 deletion (rs769765211) have the lowest plasma ACE2 level (3.52 ± 0.88 ng/mL). In the post hoc analysis, we observed that the highest (WT) and lowest (T2del) ACE2 groups differ significantly in plasma ACE2 concentration (*p* = 0.01).

We also analyzed if there is any significant difference between the mean plasma ACE2 levels between WT and the deletion variants altogether. It was observed that individuals harboring the WT variant had higher plasma ACE2 levels (5.05 ± 2.76 ng/mL) compared to individuals harboring any deletion variants (3.68 ± 0.95 ng/mL). However, the results were not statistically significant (*p* = 0.13).

**Figure 6:**
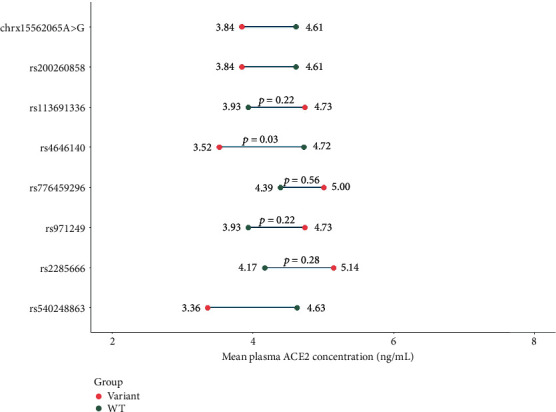
Lollipop plot showing the mean plasma ACE2 levels in individuals harboring specific ACE2 intronic variants and wild-type sequence at that particular locus. Red dots represent ACE2 levels in individual(s) harboring the specific variant, and green dots represent individuals harboring the reference sequence at that locus (three variants were identified in only one individual; therefore, the significance of the difference was not measured).

Among the 29 samples that were subjected to WES, plasma ACE2 was measured in 26 of them (6 each of control, moderate, and severe and 8 mild individuals). Apart from the 3 deletion variants on the 18T stretch, 8 other intronic variants were identified in WES. Their impact on plasma ACE2 was also analyzed. The results are shown in Figure 6.

Among the variants, only the presence of rs4646140 significantly decreases the plasma ACE2 levels (*p* = 0.03). For the rest of the variants, no significant changes in plasma ACE2 were observed. The detailed results are provided in Supplementary Table 3.

## 4. Discussion

To establish the role of host genetic variability in the progression of COVID-19, we conducted this study aimed towards exploring the genetic landscape of the *ACE2* gene in the Bangladeshi population with different degrees of COVID-19 severity and also establishing a relationship of disease severity with the levels of soluble ACE2 protein in plasma. According to dbSNP, the host receptor ACE2 (Gene ID: 59272) harbors different types of polymorphisms that include synonymous (292), noncoding transcript variant (25), inframe insertion (1), inframe deletion (6), intron (21237), and missense (701) variants (https://www.ncbi.nlm.nih.gov/snp/). Missense variants may alter the structure of the ACE2 which may affect the attachment with the spike protein of SARS-CoV-2 and, thus, disease severity.

WES and PCR-sequencing identified no genetic variant in the exons targeted in this study (exon 1, exon 2, exon 8, and exon 11). We targeted these particular regions because they encode the region of ACE2 protein that binds to the viral spike RBD [26]. According to the latest release of the gnomAD dataset (v3.1.2) (https://gnomad.broadinstitute.org), the highest allele frequencies of any missense variants in exons 1, 2, 8, and 11 are 0.4%, 0.01%, 0.4%, and 0.003%, respectively. Also, the highest frequencies of synonymous variants occurring in exons 1, 2, 8, and 11 are 0.01%, 0.01%, 0.19%, and 9.6^∗^10^−4^%, respectively. Therefore, the results are consistent with the observations of a low frequency of variants in the chromosomal regions that correspond to exons 1, 2, 8, and 11. So, observing no variants within the exonic regions can be accounted for. The low allelic frequency of missense variants is also reported by Novelli et al. [22]. Similar results were also observed in a study conducted in Turkey, where a larger cohort, consisting of 946 individuals, only revealed two missense variants (rs41303171 and rs4646116) of the *ACE2* gene in the Turkish population [33].

Also, recently, it was found that a region of our DNA situated on chromosome 3 (locus 3p21.31), spanning a length of 49.3 kb, consisting of 6 genes (SLC6A20, LZTFL1, CCR9, FYCO1, CXCR6, and XCR1), was associated with the risk of developing severe COVID-19 [34]. A later study revealed that this region was inherited from the Neanderthals and was harbored by almost half the population in South Asia [35]. The *ACE2* gene was also annotated in the Neanderthal genomes. We performed sequence alignment of the Neanderthal *ACE2* gene sequence with the human *ACE2* reference sequence and observed no changes between the *ACE2* genes from modern humans and the Neanderthal reference sequence. Indeed, individual genomes of the Neanderthals harbor a few variants when compared with that of the modern human reference, but none of those variants are in any of the exons of the *ACE2* gene. Also, our target region, the 18T stretch, was no different either. Therefore, the *ACE2* gene is highly conserved, and that is reflected in our study as well.

However, the intronic regions flanking the exons harbor many variants. Our region of interest, the 18T stretch upstream of exon 11, has shown 1, 2, or 3 T deletions (T1del, T2del, and T3del, respectively) in our study population. Although the deletion variants are not significantly associated with the disease severity, they significantly alter the plasma ACE2 level. Among the other variants, only rs4646140 is significantly associated with lower plasma ACE2 levels.

ACE2 is a tissue enzyme. Thus, circulating levels of ACE2 are low. As a result, the significance of measuring circulating ACE2 in pathological conditions remains important which may indicate a great clinical significance [36]. For example, increased levels of ACE2 have been found to be associated with an increased risk of major cardiovascular events [37, 38]. Previously, elevated levels of soluble ACE2 in the plasma of COVID-19 patients were found to be significantly associated with disease severity. Thus, the plasma ACE2 level can be a predictor of infectivity and outcome of COVID-19 [39]. We also observed a higher concentration of plasma ACE2 in the patient groups (3.94 ± 1.41 ng/mL), compared to healthy controls (3.77 ± 1.55 ng/mL). We observed the highest ACE2 concentration in the severe group of patients (4.26 ± 2 ng/mL), consolidating its ability to predict disease outcomes. However, the mean level of ACE2 in the plasma of healthy controls and patients did not vary significantly. Also, the plasma levels of soluble ACE2 in patients with mild, moderate, and severe symptoms did not show a significant difference from that of the mean values of healthy controls and among themselves as well. But the levels of soluble ACE2 in the plasma of healthy controls and mild patients were found to be lower compared to moderate and severe patients (though not significant). Maza et al. demonstrated significantly higher levels of serum ACE2 in patients with milder symptoms compared to patients with moderate and severe symptoms in the Finnish population [40]. However, in our study on the Bangladeshi population, different results were observed.

Conversely, Bani Hani et al. demonstrated that ACE2 level is elevated in critically ill patients who are admitted to the ICU, which is represented in our severe group [41]. Our results are compliant with their study. Also, in another study, Kragstrup et al. showed that high plasma ACE2 is associated with increased maximal illness severity (which corresponds to the severe group of our study) [39]. Also, the levels of ACE2 estimated in the plasma matched with that of the levels reported in Swedish individuals [42]. So, the role of plasma ACE2 is ambiguous till now, and we report in favor of its higher concentration being related to disease severity.

As reported by Iyer et al., the deletion variants, rs11340646 (T1del), rs769765211 (T2del), and rs775397699 (T3del), were not significantly associated with COVID-19 risk or disease progression [43]. Our study also did not find any significant association between the deletion variants and disease severity. Therefore, our study is in concordance with the study from Iyer et al. [43]. Also, we tried to identify if any of the variants play a protective or risk role in the COVID-19 severity, but no significant results were obtained.

The distribution pattern of soluble ACE2 in the plasma of individuals harboring T1del, T2del, and T3del has also been studied. We report the association between the T-deletion (T-del) variants and subsequent plasma ACE2 levels. The WT variant is associated with the highest plasma ACE2 level in our population, whereas T2 del is associated with the lowest level. And noticeably, the T1del and T3del variants are also associated with lower mean ACE2 levels than the wild type. To our knowledge, the association between the T-del variants with plasma ACE2 level has not been studied before. Our study is the first one to report this association.

**Figure 7:**
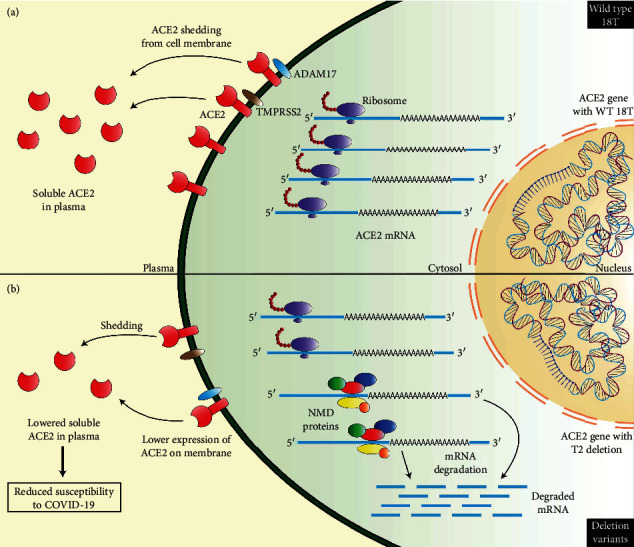
The proposed mechanism of the protective role played by the 18T deletion variants by nonsense-mediated mRNA decay (NMD). (a) The *ACE2* gene with 18T (WT) and normal expression of the ACE2 receptor. (b) The *ACE2* gene with T2del. The transcripts are targeted by NMD protein, resulting in mRNA decay and lower expression of ACE2.

The T-del variants have been identified as nonsense-mediated mRNA decay (NMD) variants in the Ensembl database that act in the surveillance mechanism in erroneous gene expression in eukaryotes [44]. This might be the possible molecular mechanism behind the lower plasma ACE2 levels in individuals harboring these variants. The presence of such variants initiates the binding of several factors to the primary transcript and will cause the elimination of premature mRNA [45]. It is evident that mutation-, codon-, gene-, cell-, and tissue-specific differences in NMD efficiency can influence the underlying disease pathology [46]. The deletion variants, on the other hand, showed a protective role against the development of severe COVID-19, as depicted by lower odds ratio. Although the results were not statistically significant, the odds ratio shows a trend of higher occurrence of the deletion variants in participants in the protective group (control and mild patients), compared to the risk group (moderate and severe patients). In fact, in the protective group consisting of 64 individuals, 57 (89%) harbored one of the deletion variants, while in the risk group (*n* = 44), 36 individuals (82%) harbored deletion variants. Therefore, deletion variants occur at a higher frequency in the protective group, compared to individuals in the risk group. Also, the presence of these genetic variants significantly decreases plasma ACE2 levels. Therefore, it is hypothesized that these variants cause lower expression of ACE2 in individuals who harbor them, by nonsense-mediated decay of the transcript. And the lower plasma ACE2 is associated with protection against the development of severe COVID-19. Our data also show higher level of plasma ACE2 in the patient group, compared to healthy controls. Also, among the patient group, severe patients had the highest plasma ACE2 level. Therefore, the presence of these NMD variants reduces plasma ACE2 level and confers protection against development of severe COVID-19. The proposed mechanism is summarized on Figure 7.

It is worth noting, however, that the regulation of gene expression in eukaryotes is achieved via a complex network. The intricate network between many processes ultimately regulates the level of expression. The presence of the NMD variants may contribute to the mechanisms that cause a decrease in gene expression, but other factors, even the presence of other variants, may also act to counteract the effect of this process. In our study, we observed the splice site variant rs2285666 occurring in 15 samples (51%) that were subjected to whole exome sequencing, out of a total of 29. This variant is reported to increase *ACE2* expression, as we have also shown in Figure 6 [47]. Also, other noncoding regions that have not been explored in this study, especially the intronic variants that are excluded while analyzing the exome, may have a significant impact on the *ACE2* expression levels. Also, the role of tissue-specific miRNAs in modulating ACE2 levels has been reported as well [48]. Another interesting observation has been made on the role of NMD in X chromosome dosage compensation and stability of transcripts originating from the X chromosome compared to autosomal transcripts [49]. In that study, it was revealed that the transcripts of the genes residing on the X chromosome have a significantly higher half-life compared to autosomal transcripts, and this feature is achieved by the contribution of UPF1, a key player of the NMD machinery. This might also explain why the presence of the NMD variants does not completely eliminate the ACE2 transcripts but rather acts as a point in the regulatory pathway. Therefore, these NMD variants might work in favor of reducing the ACE2 mRNA level but do not eliminate the transcript entirely, resulting in the expression of the protein, but only causing a decrease in the process.

Thus, noncoding intronic variants may have an impact on the expression level of the *ACE2* gene which in turn may be one of the risk-associated factors for disease severity. However, further laboratory experiments including transcriptomic studies, as well as protein level measurement in tissues, are warranted to reveal the role of these variants in the molecular mechanisms of the expression and regulation of plasma ACE2.

## 5. Conclusion

COVID-19 patients, particularly the severe group, have higher plasma ACE2 levels, compared to healthy controls. Deletion on an 18T stretch upstream of exon 11 of the ACE2 gene significantly lowers the plasma ACE2 levels. However, the deletion variants are not significantly associated with the COVID-19 disease severity.

## Figures and Tables

**Table 1:** Demographic and biochemical data of all patients of the study.

Parameter	Reference range	All patients (mean ± SD)	Stratified to severity			*p* value	Stratified to gender		*p* value
			Severe (*n* = 24)	Moderate (*n* = 20)	Mild (*n* = 24)		Male (*n* = 37)	Female (*n* = 31)	
Age (years)	—	56.1 ± 16.5	56.27 ± 14.82	61.95 ± 11.48	51.04 ± 20.89	0.09	58.1 ± 14.9	53.7 ± 18.3	0.29
Ferritin (ng/mL)	20-336	500.72 ± 524.39	607.64 ± 452.25	479.01 ± 649.72	386.45 ± 469.60	0.49	541.38 ± 460.26	461.75 ± 586.61	0.61
D-dimer (*μ*g/mL)	<0.5	1.32 ± 1.17	1.25 ± 0.95	1.86 ± 1.57	0.9 ± 0.76	0.06	1.43 ± 1.35	1.19 ± 0.93	0.49
CRP (mg/L)	<6	60.25 ± 54.80	77.825 ± 50.27	92.07 ± 57.41	15.97 ± 19.84	1.58^∗^ 10^−5^	72.29 ± 60.08	42.4 ± 41.01	0.03
O_2_ level (%)	>95	93.1 ± 5.52	88.4 ± 5.26	94.71 ± 4.27	97.4 ± 1.52	0.005	92.50 ± 6.40	93.78 ± 4.63	0.62

**Table 2:** Frequency of different variants of the *ACE2* gene observed in the WES dataset and different databases.

rs ID	Chr	Start	End	Ref	Alt	Allele frequencies (MAF) (%)		
						WES data	1000GP (phase 3)	gnomAD v3.1.2
.	chrX	15562065	15562065	A	G	3.5	N/A	N/A
rs11340646	chrX	15573562	15573562	T	—	44.8	67	56.1
rs769765211	chrX	15573562	15573563	TT	—	6.9	0.1	0.1
rs775397699	chrX	15573562	15573564	TTT	—	10.3	0.008	N/A
rs200260858	chrX	15575575	15575576	TG	—	3.5	0.7	1.8^∗^10^−3^
rs113691336	chrX	15578020	15578020	—	ATAAG	86.2	83	73
rs4646140	chrX	15587729	15587729	C	T	10.3	6	3.6
rs776459296	chrX	15589463	15589463	—	T	31	6	0.3
rs971249	chrX	15589527	15589527	T	C	86.2	80.5	69.2
rs2285666	chrX	15592225	15592225	C	T	51.7	35	23.8
rs540248863	chrX	15571886	15571886	T	G	3.5	0.5	0.1

1000 gp = 1000 Genomes Project; gnomAD = Genome Aggregation Database.

**Table 3:** Odds ratio of risk for identified variants.

Variant	Protective (*n* = 64)	Risk (*n* = 44)	*p* value	OR at 95% CI
WT	7	8	—	1
Variants				
rs11340646 (T1del)	18	16	0.68	0.78 (0.22-2.72)
rs769765211 (T2del)	36	18	0.16	0.44 (0.13-1.45)
rs775397699 (T3del)	3	2	0.60	0.61 (0.05-5.17)

## Data Availability

All data has been presented in the manuscript.

## Supplementary Materials

Supplementary Materials Supplementary Table 1: primers used to amplify the target exons and intronic region. Supplementary Table 2: PCR conditions used to amplify the target regions. Supplementary Table 3: mean ACE2 level in individuals harboring specific ACE2 intronic variants or wild-type variants at a particular locus. Supplementary Figure 1: gel electrophoresis of different amplicons. Three distinct bands consisting of 468 base pairs for exon 1, 432 base pairs for exon 2, and 500 base pairs for exon 8 have been presented in the figure. NC: negative control and a 100-base pair DNA ladder were used as a marker. Supplementary Figure 2: gel electrophoresis of amplicons consisting of 900 bp. The noncoding region covering the stretch of 18 “T” nucleotides along with exon 11 of the ACE2 receptor gene. Distinct bands consisting of 900 base pairs have been identified. NC: negative control and a 100-base pair DNA ladder were used as a marker.

## References

[1] Zhou R., Li F., Chen F. (2020). Viral dynamics in asymptomatic patients with COVID-19. *International Journal of Infectious Diseases*.

[2] Wang M.-Y., Zhao R., Gao L.-J., Gao X.-F., Wang D.-P., Cao J.-M. (2020). SARS-CoV-2: structure, biology, and structure-based therapeutics development. *Frontiers in Cellular and Infection Microbiology*.

[3] Bourgonje A. R., Abdulle A. E., Timens W. (2020). Angiotensin-converting enzyme 2 (ACE2), SARS-CoV-2 and the pathophysiology of coronavirus disease 2019 (COVID-19). *Journal of Pathology*.

[4] Naqvi A. A. T., Fatima K., Mohammad T. (2020). Insights into SARS-CoV-2 genome, structure, evolution, pathogenesis and therapies: structural genomics approach. *Biochimica et Biophysica Acta - Molecular Basis of Disease*.

[5] Venkataraman S., Prasad B. V. L. S., Selvarajan R. (2018). RNA dependent RNA polymerases: insights from structure, function and evolution. *Viruses*.

[6] Mukherjee R., Satardekar R. (2021). Why are some coronavirus variants more infectious?. *Journal of Biosciences*.

[7] Onabajo O. O., Banday A. R., Stanifer M. L. (2020). Interferons and viruses induce a novel truncated ACE2 isoform and not the full-length SARS-CoV-2 receptor. *Nature Genetics*.

[8] Salamanna F., Maglio M., Landini M. P., Fini M. (2020). Body localization of ACE-2: on the trail of the keyhole of SARS-CoV-2. *Frontiers in Medicine*.

[9] Chaudhary M. (2020). COVID-19 susceptibility: potential of ACE2 polymorphisms. *Egyptian Journal of Medical Human Genetics*.

[10] Nabi A. H. M. N., Ebihara A., McFarlane S. I. (2021). Diabetes and renin-angiotensin-aldosterone system: pathophysiology and genetics. *Renin-Angiotensin Aldosterone System*.

[11] South A. M., Tomlinson L., Edmonston D., Hiremath S., Sparks M. A. (2020). Controversies of renin–angiotensin system inhibition during the COVID-19 pandemic. *Nature Reviews Nephrology*.

[12] Cao Y., Li L., Feng Z. (2020). Comparative genetic analysis of the novel coronavirus (2019-nCoV/SARS-CoV-2) receptor ACE2 in different populations. *Cell Discovery*.

[13] Pan Y., Wang T., Li Y. (2018). Association of ACE2 polymorphisms with susceptibility to essential hypertension and dyslipidemia in Xinjiang, China. *Lipids in Health and Disease*.

[14] Patnaik M., Pati P., Swain S. N. (2014). Association of angiotensin-converting enzyme and angiotensin-converting enzyme-2 gene polymorphisms with essential hypertension in the population of Odisha, India. *Annals of Human Biology*.

[15] García L. F. (2020). Immune response, inflammation, and the clinical spectrum of COVID-19. *Frontiers in Immunology*.

[16] Taylor C. A., Whitaker M., Anglin O. (2022). COVID-19–associated hospitalizations among adults during SARS-CoV-2 delta and omicron variant predominance, by race/ethnicity and vaccination status—COVID-NET, 14 states, July 2021–January 2022. *Morbidity and Mortality Weekly Report*.

[17] Weaver A. K., Head J. R., Gould C. F., Carlton E. J., Remais J. V. (2022). Environmental factors influencing COVID-19 incidence and severity. *Annual Review of Public Health*.

[18] Pojero F., Candore G., Caruso C. (2021). The role of immunogenetics in COVID-19. *International Journal of Molecular Sciences*.

[19] Cao W., Chen C., Li M. (2021). Important factors affecting COVID-19 transmission and fatality in metropolises. *In Public Health*.

[20] Gómez J., Albaiceta G. M., García-Clemente M. (2020). Angiotensin-converting enzymes (ACE, ACE2) gene variants and COVID-19 outcome. *Gene*.

[21] Karakaş Çelik S., Çakmak Genç G., Pişkin N. (2021). Polymorphisms of ACE (I/D) and ACE2 receptor gene (Rs2106809, Rs2285666) are not related to the clinical course of COVID-19: a case study. *Journal of Medical Virology*.

[22] Novelli A., Biancolella M., Borgiani P. (2020). Analysis of ACE2 genetic variants in 131 Italian SARS-CoV-2-positive patients. *Human Genomics*.

[23] Sienko J., Marczak I., Kotowski M. (2022). Association of ACE2 gene variants with the severity of COVID-19 disease-a prospective observational study. *International Journal of Environmental Research and Public Health*.

[24] Cafiero C., Rosapepe F., Palmirotta R. (2021). Angiotensin system polymorphisms’ in SARS-CoV-2 positive patients: assessment between symptomatic and asymptomatic patients: a pilot study. *Pharmacogenomics and Personalized Medicine*.

[25] Suryamohan K., Diwanji D., Stawiski E. W. (2021). Human ACE2 receptor polymorphisms and altered susceptibility to SARS-CoV-2. *Communications Biology*.

[26] Tahsin A., Ahmed R., Bhattacharjee P. (2022). Most frequently harboured missense variants of hACE2 across different populations exhibit varying patterns of binding interaction with spike glycoproteins of emerging SARS-CoV-2 of different lineages. *Computers in Biology and Medicine*.

[27] Yuki K., Fujiogi M., Koutsogiannaki S. (2020). COVID-19 pathophysiology: a review. *Clinical Immunology*.

[28] Bappy H. M. J. A., Goswami A., Huda N., Hosen M. I., Nabi A. H. M. N. (2020). Gender specific association of missense variant rs1805097 of IRS-2 and noncoding variant rs841853 of GLUT-1 genes with susceptibility to type 2 diabetes in Bangladeshi population. *Gene Reports*.

[29] Goswami A., Huda N., Yasmin T., Hosen M. I., Hasan A. K. M. M., Nabi A. H. M. N. (2021). Association study of leukocyte telomere length and genetic polymorphism within hTERT promoter with type 2 diabetes in Bangladeshi population. *Molecular Biology Reports*.

[30] Huda N., Hosen M. I., Yasmin T., Sarkar P. K., Hasan A. K. M. M., Nabi A. H. M. N. (2018). Genetic variation of the transcription factor GATA3, not STAT4, is associated with the risk of type 2 diabetes in the Bangladeshi population. *PLoS One*.

[31] Wickham H. (2016). *ggplot2: Elegant Graphics for Data Analysis*.

[32] Wickham H., François R., Henry L., Müller K. (2022). dplyr: A grammar of data manipulation. https://github.com/tidyverse/dplyr.

[33] Duman N., Tuncel G., Bisgin A. (2022). Analysis of ACE2 and TMPRSS2 coding variants as a risk factor for SARS-CoV-2 from 946 whole-exome sequencing data in the Turkish population. *Journal of Medical Virology*.

[34] Genomewide Association Study of Severe Covid-19 with Respiratory Failure (2020). Genomewide association study of severe Covid-19 with respiratory failure. *England Journal of Medicine*.

[35] Zeberg H., Pääbo S. (2020). The major genetic risk factor for severe COVID-19 is inherited from Neanderthals. *Nature*.

[36] Tikellis C., Johnston C. I., Forbes J. M. (2003). Characterization of renal angiotensin-converting enzyme 2 in diabetic nephropathy. *Hypertension*.

[37] Fagyas M., Fejes Z., Sütő R. (2022). Circulating ACE2 activity predicts mortality and disease severity in hospitalized COVID-19 patients. *International Journal of Infectious Diseases*.

[38] Nagy B. J., Fejes Z., Szentkereszty Z. (2021). A dramatic rise in serum ACE2 activity in a critically ill COVID-19 patient. *International Journal of Infectious Diseases*.

[39] Kragstrup T. W., Singh H. S., Grundberg I. (2021). Plasma ACE2 predicts outcome of COVID-19 in hospitalized patients. *PLoS One*.

[40] Maza M. D. C., Úbeda M., Delgado P. (2022). ACE2 serum levels as predictor of infectability and outcome in COVID-19. *Frontiers in Immunology*.

[41] Bani Hani A., Abu Tarboush N., Bani Ali M. (2022). Serum ACE2 level is associated with severe SARS-CoV-2 infection: a cross-sectional observational study. *Biomarker Insights*.

[42] Lundström A., Ziegler L., Havervall S. (2021). Soluble angiotensin-converting enzyme 2 is transiently elevated in COVID-19 and correlates with specific inflammatory and endothelial markers. *Journal of Medical Virology*.

[43] Iyer G. R., Samajder S., Zubeda S. (2020). Infectivity and progression of COVID-19 based on selected host candidate gene variants. *Frontiers in Genetics*.

[44] Kurosaki T., Popp M. W., Maquat L. E. (2019). Publisher correction: quality and quantity control of gene expression by nonsense-mediated mRNA decay. *Nature reviews. Molecular Cell Biology*.

[45] Dyle M. C., Kolakada D., Cortazar M. A., Jagannathan S. (2020). How to get away with nonsense: mechanisms and consequences of escape from nonsense-mediated RNA decay. *Wiley Interdisciplinary Reviews: RNA*.

[46] Khajavi M., Inoue K., Lupski J. R. (2006). Nonsense-mediated mRNA decay modulates clinical outcome of genetic disease. *European Journal of Human Genetics: EJHG*.

[47] Vadgama N., Kreymerman A., Campbell J. (2022). SARS-CoV-2 susceptibility and ACE2 gene variations within diverse ethnic backgrounds. *Frontiers in Genetics*.

[48] Widiasta A., Sribudiani Y., Nugrahapraja H., Hilmanto D., Sekarwana N., Rachmadi D. (2020). Potential role of ACE2-related microRNAs in COVID-19-associated nephropathy. *Non-Coding RNA Research*.

[49] Faucillion M.-L., Larsson J. (2015). Increased expression of X-linked genes in mammals is associated with a higher stability of transcripts and an increased ribosome density. *Genome Biology and Evolution*.

