# Supplementary material for: Intronic Variants of the Angiotensin-Converting Enzyme 2 Gene Modulate Plasma ACE2 Levels and Possibly Confer Protection against Severe COVID-19
Source: Biomed Res Int. 2023 Oct 26;2023:5705076. doi: 10.1155/2023/5705076 (PMC10622595; doi:10.1155/2023/5705076)

**Supplementary Table 1:** Primers used to amplify the target exons and intronic region.

| Set | Target | Direction | Sequence | Length (bp) | Tm  (°C) | Product size (bp) |
| --- | --- | --- | --- | --- | --- | --- |
| 1 | 18T  Stretch  with exon 11 | Forward  (5’-3’) | GTGTGGTGCCTGAGAGGAAT | 20 | 61.86 | 900 |
|  |  | Reverse  (5’-3’) | TTGAGCTTGGTAGTTATGGGTGG | 23 | 60.24 |  |
| 2 | Exon 1 | Forward  (5’-3’) | GGCTCAGCAGATTGTTTACTGTG | 23 | 60.12 | 468 |
|  |  | Reverse  (5’-3’) | GGCAAACATCCAATCTCACAACT | 23 | 59.74 |  |
| 3 | Exon 2 | Forward  (5’-3’) | AACCTTGACCTTCAGCGGAG | 20 | 59.96 | 432 |
|  |  | Reverse  (5’-3’) | GAGAGAGATTTTTCTAACATCAGCA | 25 | 57.16 |  |
| 4 | Exon 8 | Forward  (5’-3’) | CCACCCCATAGCCTCTGTAA | 20 | 59.95 | 500 |
|  |  | Reverse  (5’-3’) | AACCCAAATGTGCTCTCCTG | 20 | 60.11 |  |

FP= forward primer, RP= reverse primer, bp= base pair

**Supplementary Table 2**: PCR Conditions used to amplify the target regions.

| Primer Set | Tm  ( °C) | PCR conditions | | | | | |
| --- | --- | --- | --- | --- | --- | --- | --- |
|  |  | 95°C  (Minutes) | Thermal cycle (40 cycles) | | | 72°C  (Minutes) |  |
|  |  |  | 95°C (seconds) | Tm (seconds) | 72°C (seconds) |  | 4°C (Seconds) |
| 1 | 61 | 5 | 45 | 42 | 95 | 5 | Hold |
| 2 | 59 | 5 | 45 | 42 | 95 | 5 | Hold |
| 3 | 59 | 5 | 45 | 42 | 95 | 5 | Hold |
| 4 | 59 | 5 | 45 | 42 | 95 | 5 | Hold |

**Supplementary Table 3:** Mean ACE2 level in individuals harboring specific ACE2 intronic variants or wild-type variants at a particular locus.

| Variants | No. of Samples  with this variant | Variant | WT | P value |
| --- | --- | --- | --- | --- |
|  |  | Mean ± SD (ng/mL) | Mean ± SD (ng/mL) |  |
| rs113691336 | 21 | 4.73 ± 2.09 | 3.93 ± 0.96 | 0.2221 |
| rs776459296 | 8 | 5.00 ± 2.67 | 4.39 ± 1.57 | 0.5657 |
| rs971249 | 21 | 4.73 ± 2.09 | 3.93 ± 0.96 | 0.2221 |
| rs2285666 | 11 | 5.14 ± 2.76 | 4.17 ± 0.911 | 0.2879 |
| rs4646140 | 3 | 3.52 ± 0.495 | 4.72 ± 2.02 | **0.03391** |
| chrx15562065A>G | 1 | 3.84 | 4.61 ± 1.97 | NA |
| rs200260858 | 1 | 3.84 | 4.61 ± 1.97 | NA |
| rs540248863 | 1 | 3.36 | 4.63 ± 1.96 | NA |

**Supplementary Figure 1:** Gel electrophoresis of different amplicons. Three distinct bands consisting of 468 base pairs for exon 1, 432 base pairs for exon 2, and 500 base pairs for exon 8 have been presented in the figure. NC: Negative control and a 100-base pair DNA ladder were used as markers.


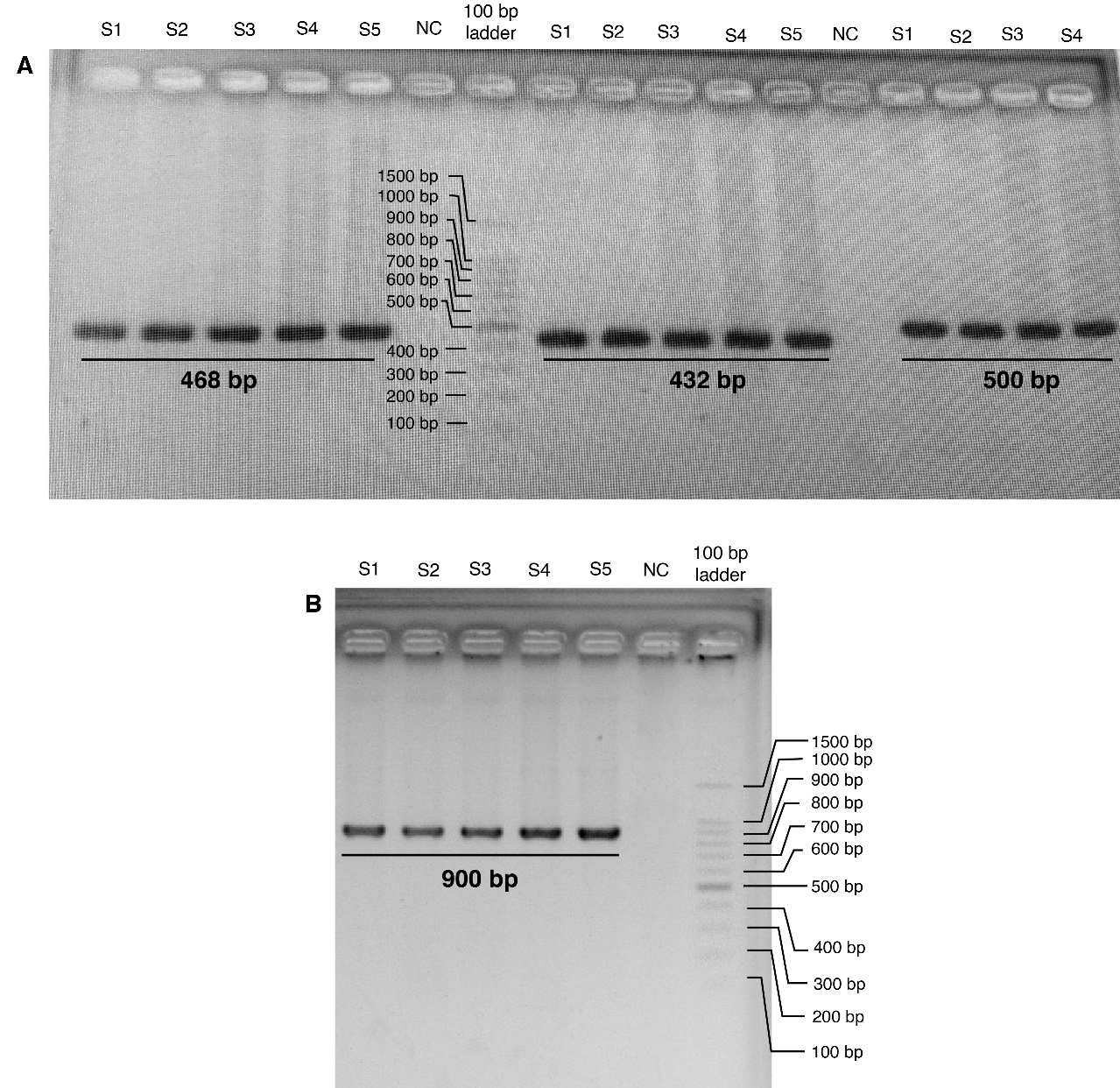


**Supplementary Figure 2:** Gel electrophoresis of amplicons consisting of 900 bp. The non-coding region covering the stretch of 18 ‘T’ nucleotides along with exon 11 of the hACE2 receptor gene. Distinct bands consisting of 900 base pairs have been identified. NC: Negative control and a 100-base pair DNA ladder were used as a marker.


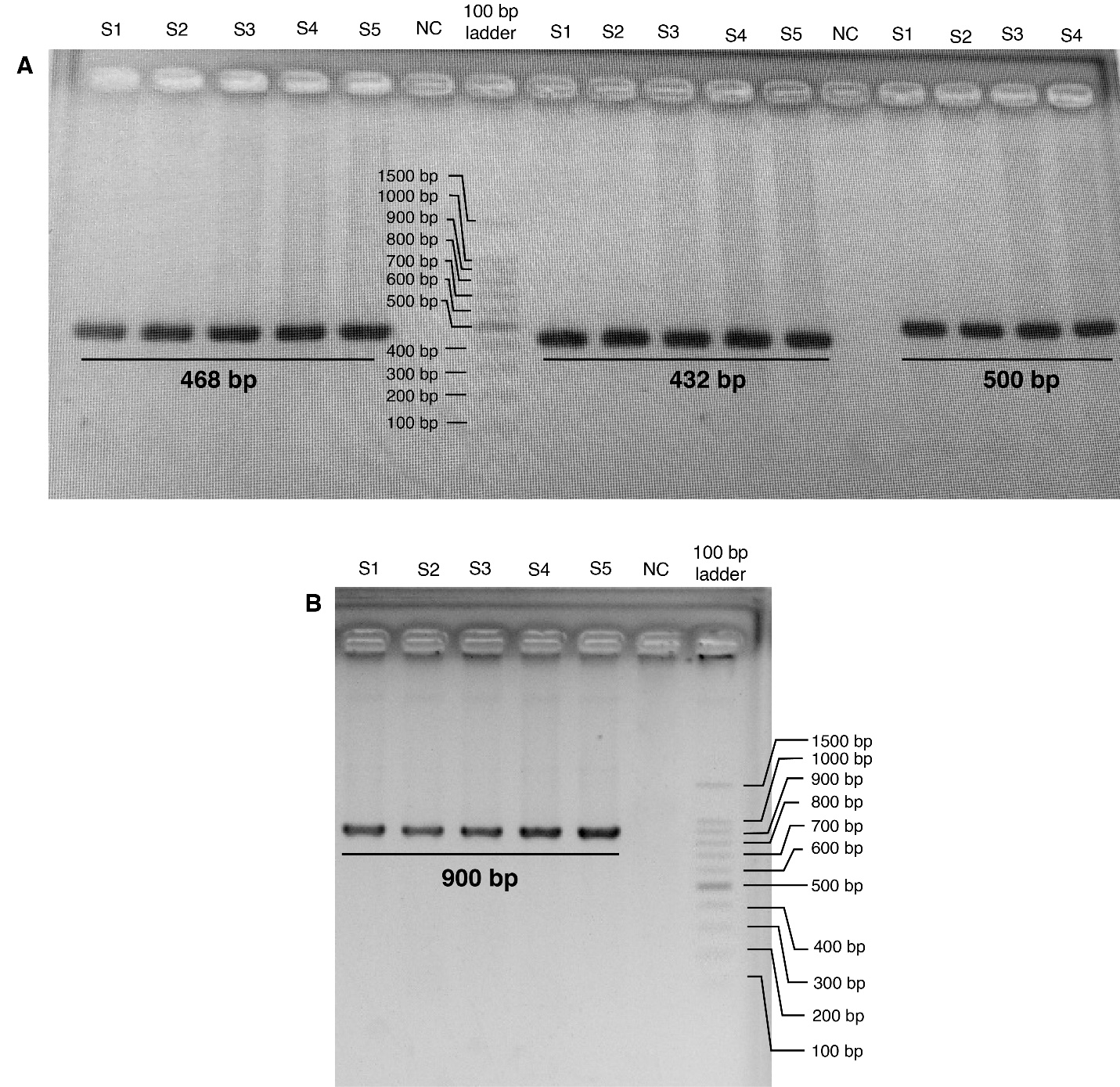

Supplement: Supplementary Materials — Supplementary Table 1: primers used to amplify the target exons and intronic region. Supplementary Table 2: PCR conditions used to amplify the target regions. Supplementary Table 3: mean ACE2 level in individuals harboring specific ACE2 intronic variants or wild-type variants at a particular locus. Supplementary Figure 1: gel electrophoresis of different amplicons. Three distinct bands consisting of 468 base pairs for exon 1, 432 base pairs for exon 2, and 500 base pairs for exon 8 have been presented in the figure. NC: negative control and a 100-base pair DNA ladder were used as a marker. Supplementary Figure 2: gel electrophoresis of amplicons consisting of 900 bp. The noncoding region covering the stretch of 18 “T” nucleotides along with exon 11 of the ACE2 receptor gene. Distinct bands consisting of 900 base pairs have been identified. NC: negative control and a 100-base pair DNA ladder were used as a marker. [file 5705076.f1.docx]
